# The Misuse of Hospitals

**Published:** 1895-07-27

**Authors:** 


### The Misuse of Hospitals.—III.

### POINTS FOR THE PROPOSED CONFERENCE.

We have before had occasion to point out that it has been contended by those who have essayed to speak on behalf of the general practitioner of medicine, that the admission of paying patients to a general hospital is an abuse of its privileges and must be withstood to the death. Such contentions are due to misapprehension, for rightly understood and properly applied the pay system should prove a bulwark for the general practitioner, and should tend more to protect his interests by abolishing the abuse of hospitals than anything which can be mentioned. The misapprehension is due to a belief on the part of the general practitioners that the admission of paying patients to a general hospital is the same thing as the taking of payment from a patient in the general wards of a hospital. Now, rightly understood, these two things are wholly apart and distinct from each other. It is impossible for any general hospital in this country to accept payment from a patient in a general ward, without doing injustice to the hospital and also to the general practitioner of medicine. This is so because any hospital which takes payment from the patients it admits for treatment, must provide in its rules that a certain proportion of these payments shall be devoted towards the cost of medical attendance. When, however, a hospital sets apart special buildings or wards for the sole treatment of pay patients, the pay system must prove a protection both to the institution and to the general practitioner of medicine, providing always that the rules of admission are properly drawn and enforced. In the latter circumstances every pay patient in the pay wing might be properly and rightly treated by his own medical man, in consultation with the staff of the hospital, upon terms which would prove mutually satisfactory. Here then is the root of the whole difficulty which the proposed conferences must consider fully and adequately, with a view to arriving at a wise solution of the present most objectionable system.

Apart from the question of payment for the medical treatment of the in-patients at the voluntary hospitals, on a plan which would be just to all the members of the medical profession and satisfactory to the public, there is the further question of the out-patients. The numbers of out-patients which attend at the hospitals in London are increasing by leaps and bounds. The better the accommodation of the out-patient department, the more thoroughly it is organised, and the higher the scale of the medical attendance, the greater becomes its popularity and the number of people who flock to it for medical treatment. We are of opinion that the conference should consider the present state of affairs very seriously on their merits. It has been patent to us for some years that the hospitals will never do this branch of their work satisfactorily until they enter into an arrangement with the general practitioners, and also with the workpeople at the manufactories and establishments from which the patients come. The Hospital Saturday Fund has done service to the hospitals by finding out, classifying, and publishing the names and addresses of the chief firms and employers of labour in London. We are of opinion that out-patient registers at the hospitals should contain columns for the insertion of the name and address of each patient, and the name and address of his employer, and the date when he was last at work. In this way they would be able to send in to each workshop to the employer, for the information of the employed, a complete statement of the number of patients received from it and the cost of their treatment. They would then gradually be able to induce the more intelligent and thrifty of the working classes to institute a regular system of contributions to the hospital box in each workshop. Such a system would give the foremen funds, out of which they might, in the first instance, defray the fee of the local medical attendant, whose services the employé might call in; and then, but only then if the nature of the case warranted it, the patient could be sent forward to the hospital as an out-patient.

